# Clinical and molecular characterisation of hereditary dopamine transporter deficiency syndrome: an observational cohort and experimental study

**DOI:** 10.1016/S1474-4422(10)70269-6

**Published:** 2010-11-26

**Authors:** Manju A Kurian, Yan Li, Juan Zhen, Esther Meyer, Nebula Hai, Hans-Jürgen Christen, Georg F Hoffmann, Philip Jardine, Arpad von Moers, Santosh R Mordekar, Finbar O'Callaghan, Evangeline Wassmer, Elizabeth Wraige, Christa Dietrich, Timothy Lewis, Keith Hyland, Simon JR Heales, Terence Sanger, Paul Gissen, Birgit E Assmann, Maarten EA Reith, Eamonn R Maher

**Affiliations:** a Department of Medical and Molecular Genetics, University of Birmingham School of Medicine, Institute of Biomedical Research, and Centre for Rare Diseases and Personalised Medicine, University of Birmingham, Birmingham, UK; b Department of Paediatric Neurology, Birmingham Children's Hospital, Birmingham, UK; c Department of Psychiatry and Pharmacology, Millhauser Laboratories, New York University School of Medicine, New York, NY, USA; d Children's Hospital Auf der Bult, Hannover, Germany; e Department of General Pediatrics, University of Heidelberg, Heidelberg, Germany; f Department of Paediatric Neurology, Bristol Children's Hospital, Bristol, UK; g DRK Kliniken Berlin Westend, Children's Hospital, Berlin, Germany; h Department of Paediatric Neurology, Sheffield Children's Hospital, Sheffield, UK; i Department of Paediatric Neurology, Evelina Children's Hospital, London, UK; j Department of Radiology, Frenchay Hospital, Bristol, UK; k Department of Neuroscience, Bristol University, Bristol, UK; l Medical Neurogenetics, Atlanta, GA, USA; m Neurometabolic Unit, National Hospital and Department of Chemical Pathology, Great Ormond Street Hospital, London, UK; n University of Southern California, Department of Biomedical Engineering, Los Angeles, CA, USA; o University Children's Hospital, Department of General Paediatrics, Dusseldorf, Germany; p West Midlands Regional Genetics Service, Birmingham Women's Hospital, Birmingham, UK

## Abstract

**Background:**

Dopamine transporter deficiency syndrome is the first identified parkinsonian disorder caused by genetic alterations of the dopamine transporter. We describe a cohort of children with mutations in the gene encoding the dopamine transporter (*SLC6A3*) with the aim to improve clinical and molecular characterisation, reduce diagnostic delay and misdiagnosis, and provide insights into the pathophysiological mechanisms.

**Methods:**

11 children with a biochemical profile suggestive of dopamine transporter deficiency syndrome were enrolled from seven paediatric neurology centres in the UK, Germany, and the USA from February, 2009, and studied until June, 2010. The syndrome was characterised by detailed clinical phenotyping, biochemical and neuroradiological studies, and *SLC6A3* mutation analysis. Mutant constructs of human dopamine transporter were used for in-vitro functional analysis of dopamine uptake and cocaine-analogue binding.

**Findings:**

Children presented in infancy (median age 2·5 months, range 0·5–7) with either hyperkinesia (n=5), parkinsonism (n=4), or a mixed hyperkinetic and hypokinetic movement disorder (n=2). Seven children had been initially misdiagnosed with cerebral palsy. During childhood, patients developed severe parkinsonism-dystonia associated with an eye movement disorder and pyramidal tract features. All children had raised ratios of homovanillic acid to 5-hydroxyindoleacetic acid in cerebrospinal fluid, of range 5·0–13·2 (normal range 1·3–4·0). Homozygous or compound heterozygous *SLC6A3* mutations were detected in all cases. Loss of function in all missense variants was recorded from in-vitro functional studies, and was supported by the findings of single photon emission CT DaTSCAN imaging in one patient, which showed complete loss of dopamine transporter activity in the basal nuclei.

**Interpretation:**

Dopamine transporter deficiency syndrome is a newly recognised, autosomal recessive disorder related to impaired dopamine transporter function. Careful characterisation of patients with this disorder should provide novel insights into the complex role of dopamine homoeostasis in human disease, and understanding of the pathophysiology could help to drive drug development.

**Funding:**

Birmingham Children's Hospital Research Foundation, Birth Defects Foundation Newlife, Action Medical Research, US National Institutes of Health, Wellchild, and the Wellcome Trust.

## Introduction

Dopaminergic neurons expressing the dopamine transporter (DAT) are located predominantly in the substantia nigra pars compacta, with projections to the striatum via the nigrostriatal pathway, and in the ventral tegmental area of the midbrain, with mesocorticolimbic projections to the nucleus accumbens, hippocampus, and other corticolimbic structures.^1^ Consistent with this cerebral distribution, dopamine has a wide variety of important physiological functions including motor control, cognition, and behaviour.^2^, ^3^, ^4^ Defects in the dopamine biosynthetic pathway (webappendix p 1) result in complex, predominantly extrapyramidal neurological disorders.^5^ DAT has an important homoeostatic role in dopaminergic transmission, and variants of the gene encoding DAT (*SLC6A3*) and other factors causing dopamine dysregulation have been implicated in several neurological and neuropsychiatric diseases.^6^, ^7^

A syndrome of infantile parkinsonism-dystonia and raised dopamine metabolites in cerebrospinal fluid (CSF) has been described,^8^ and we have previously reported three children with this phenotype in whom we identified loss-of-function mutations in *SLC6A3* (gene locus 5p15.3).^9^ To delineate this novel disorder, dopamine transporter deficiency syndrome, we describe the clinical, biochemical, molecular genetic, and functional aspects of 11 children with germline *SLC6A3* mutations.

## Methods

### Patients

11 children with suspected dopamine transporter deficiency syndrome were enrolled from seven paediatric neurology centres in the UK, Germany, and the USA. These patients were identified by contact with paediatric neurologists specialising in movement disorders or neurotransmitter diseases, and liaising with specialist laboratories doing paediatric CSF neurotransmitter analysis (Institute of Child Health, London, UK; Medical Neurogenetics, Atlanta, GA, USA; University Children's Hospital, Heidelberg, Germany; Kinderspital, Zurich, Switzerland). Potential patients were also identified by searching PubMed for published clinical cases. Patients were enrolled into the study from February, 2009, onwards and the clinical phenotype was assessed at regular intervals thereafter; the cutoff date for inclusion of data in this report was June, 2010. A brief overview of the clinical phenotype of patients 1–3 was reported in association with the initial identification of *SLC6A3* mutations in 2009,^9^ and the clinical features of patients 4–6 were briefly reported in 2004.^8^ The study research protocol was approved by local research ethics committees and written informed consent was obtained for participating individuals from their next of kin.

### Procedures

All children were clinically assessed by a paediatric neurologist. Video footage documenting the clinical features of patients from early infancy to present time was obtained either by their parents (in their home environment) or by the examining paediatric neurologist (during a clinic or hospital appointment). The duration of video footage obtained, and the intervals between video recordings, varied between patients. Acquired clinical videos were reviewed independently by three paediatric neurologists in June, 2010. Medical case notes were reviewed to establish the clinical history, pattern of disease evolution, and response to drug treatment.

CSF neurotransmitter analysis was done in all patients, and concentrations of homovanillic acid and 5-hydroxyindoleacetic acid were measured by use of laboratory-specific age-related reference ranges.^10^, ^11^, ^12^ Homovanillic acid and 5-hydroxyindoleacetic acid are the stable degradation products of dopamine and serotonin, respectively, and thus are indicative of the turnover of dopamine and serotonin.^13^, ^14^ Urine catecholamine metabolites, serum prolactin, and serum creatine kinase were analysed. We also did neuroradiological studies: MRI in all patients; magnetic resonance spectroscopy (MRS) in patients 1, 2, and 11; and nuclear brain imaging with single photon emission CT DaTSCAN (GE Healthcare, Amersham, UK) in patient 3 after administration of the DAT ligand ioflupane (^123^I-2β-carbometoxy-3β-[4-iodophenyl]-N-[3-fluoropropyl] nortropane).

In molecular genetic studies, all annotations and physical positions were recorded as in the National Centre for Biotechnology Information's genome build (version 36.3). Analysis of the *SLC6A3* gene was done by direct sequencing as described previously.^9^ Long-range PCR was used to identify the deletion breakpoint in patient 8 (webappendix p 2).

Mutant constructs of human DAT (hDAT) were prepared from wild-type pCIN4-hDAT.^9^ The primers and their complementary primers are described in webappendix p 3. Culturing and transient transfection of HEK293 cells with mutant and wild-type hDAT was done by use of Lipofectamine 2000 (Invitrogen, Carlsbad, CA, USA) as previously described.^9^ Uptake of ^3^H-dopamine into cells expressing hDAT was measured for 5 min at 21°C, and to monitor cocaine-analogue binding, cells were incubated with 4 nM ^3^H-CFT (2β-carbomethoxy-3β-[4-fluorophenyl]-tropane) for 20 min at 21°C; for saturation analysis, 0·1–100 nM non-radioactive CFT was also present.^9^ All other methods and analyses were as described by us previously.^9^ Briefly, uptake and binding assays used a high sodium, low potassium buffer containing glucose and tropolone, and the non-specific binding was defined as 1 μM CFT. The binding affinity (K_d_) and maximum binding of ^3^H-CFT were estimated with non-linear regression by use of Radlig software (KELL program). Half maximal inhibitory concentration was estimated by logistic fitting with ORIGIN software (OriginLab), and this value was then inputted into the Cheng-Prusoff equation to calculate the potency (K_i_) of dopamine in inhibition of ^3^H-CFT binding.

### Statistical analysis

Binding results for wild-type and mutant DAT were first compared by one-way ANOVA. If this test indicated significant differences between groups, the Dunnett multiple comparisons test was used to compare each mutant value with wild type. We regarded p values of 0·05 or lower as significant. Two sets of experiments were done: wild-type DAT (controls) were compared with mutants (Leu368Gln and Pro395Leu) for patients 1–3;^9^ and wild-type DAT (controls) were compared with mutants (Pro554Leu, Gly327Arg, Gly327Arg plus Gln439X, Pro529Leu, Leu224Pro, and Arg521Trp) for patients 4–11. Val158Phe was not included in the statistical analysis because no binding was detectable. Samples consisted of three to five independent experiments for which mean (SE) was calculated; because two sets of experiments were done, we calculated the combined mean (SE) for controls from eight to nine independent experiments. In each experiment, binding assays were done in triplicate on cell preparations expressing either the wild-type or mutant DAT construct. ANOVA and Dunnett statistical tests were applied to each set separately.

### Role of the funding source

The funding sources had no role in the study design, data collection, data analysis, data interpretation, writing of the report, or the decision to submit for publication. All authors had full access to all data in the study. The corresponding author (MAK), MEAR, and ERM had final responsibility for the decision to submit for publication.

## Results

11 children (two boys, nine girls) with dopamine transporter deficiency syndrome were enrolled into the study. All children presented with a movement disorder with onset in early infancy (median age 2·5 months, range 0·5–7; table 1). Seven children were alive at the time of writing this report (age range 1·9–11·4 years). Before diagnosis, seven children had been misdiagnosed with cerebral palsy (patients 1–3, 5, and 7–9). None of the children had a family history of Parkinson's disease, or movement or neuropsychiatric disorders. Neonatal irritability and early feeding difficulties were evident in six children (patients 1–4, 8, and 11). Five children presented mainly with hyperkinetic symptoms (dystonia, chorea, dyskinesia; patients 7–11), four had predominantly hypokinetic and parkinsonian features (patients 1, 2, 5, and 6), and two (patients 3 and 4) had a mixed hyperkinetic and hypokinetic movement disorder. Axial hypotonia was evident in eight children on presentation (patients 1–5 and 8–10).

**Table 1:** Overview of clinical features of dopamine transporter deficiency syndrome

	**Sex**	**Age (years)**	**Age at clinical presentation (months)**	**Early motor features present at disease onset**				**Early motor features developing before 3 years of age**						**Motor features developing after 3 years of age**				**EMD**	**BD**	**MCP**	**Maximum ratio of HVA to HIAA**
				PHF*	Dk†	Dt	AH	PHF*	Dk†	Dt	AH	PTF	MDD‡	PHF*	Dt	PTF	MDD‡				
Patient 1	M	5·7	0·75	Yes	No	No	Yes	Yes	No	Yes	Yes	Yes	Yes	Yes	Yes	Yes	Yes	No	Yes	Yes	13·2
Patient 2	F	4·2	3	Yes	No	No	Yes	Yes	No	Yes	Yes	Yes	Yes	Yes	Yes	Yes	Yes	No	Yes	Yes	6·8
Patient 3	F	11·4	5	Yes	No	Yes	Yes	Yes	Yes	Yes	Yes	Yes	Yes	Yes	Yes	Yes	Yes	Yes	Yes	Yes	12·5
Patient 4	F	16·2 (died)	4	Yes	No	Yes	Yes	Yes	Yes	Yes	Yes	Yes	Yes	Yes	Yes	Yes	Yes	Yes	Yes	No	12·1
Patient 5	F	8·9 (died)	4	Yes	No	No	Yes	Yes	Yes	Yes	Yes	Yes	Yes	Yes	Yes	Yes	Yes	Yes	Yes	Yes	11·4
Patient 6	M	15·0 (died)	2·5	Yes	No	No	No	Yes	Yes	Yes	Yes	Yes	Yes	Yes	Yes	Yes	Yes	Yes	Yes	No	12·1
Patient 7	F	14·2 (died)	1·5	No	Yes	Yes	No	Yes	Yes	Yes	Yes	No	Yes	Yes	Yes	Yes	Yes	Yes	Yes	Yes	6·6
Patient 8	F	2·0	0·5	No	No	Yes	Yes	Yes	No	Yes	Yes	No	Yes	NA	NA	NA	NA	Yes	Yes	Yes	8·6
Patient 9	F	6·0	3	No	No	Yes	Yes	Yes	No	Yes	Yes	Yes	Yes	Yes	Yes	Yes	Yes	Yes	Yes	Yes	12·9
Patient 10	F	1·9	7	No	Yes	No	Yes	Yes§	Yes	Yes	Yes	No	Yes	NA	NA	NA	NA	Yes	Yes	No	10·5
Patient 11	F	4·3	0·5	No	No	Yes	No	Yes	No	Yes	Yes	No	Yes	Yes	Yes	No	Yes	No	Yes	No	11·0

EMD=eye movement disorder. BD=bulbar dysfunction. MCP=misdiagnosis with cerebral palsy. HVA=homovanillic acid. HIAA=5-hydroxyindoleacetic acid. PHF=parkinsonian hypokinetic features. Dk=dyskinesia. Dt=dystonia. AH=axial hypotonia. PTF=pyramidal tract features. MDD=motor developmental delay. M=male. F=female. NA=not applicable (patient younger than 3 years).

* Bradykinesia, rigidity, tremor, or hypomimia.

† Chorea or restlessness.

‡ Delay in gross motor skills.

§ Hypomimia only.

Clinical features of dopamine transporter deficiency syndrome included infantile hyperkinesia (webvideos 1 and 2), orolingual dyskinesia (webvideo 1, clip 2), dystonia (webvideo 3), parkinsonian features (webvideos 4–7), pyramidal tract features, axial hypotonia, and eye movement abnormalities (webvideo 8).

An early hyperkinetic movement disorder was seen in six patients (patients 3–7 and 10). Two patients presented with repetitive generalised choreiform movements of variable amplitude (patients 7 and 10; webvideo 2), and a further four developed choreiform movements of the lower limbs within a year of presentation (patients 3–6). As these six patients increased in age to 2–4 years, hyperkinesia reduced gradually during childhood. Orolingual dyskinesia was recorded in six children (patients 1, 3–5, 8, and 10).

Generalised dystonia was recorded in all children: in six at symptom onset (patients 3, 4, 7–9, and 11), and within 1 year of clinical presentation in the remaining children (patients 1, 2, 5, 6, and 10). All children had oromandibular dystonia, eight had bilateral striatal toe (patients 3–9 and 11), five had prominent upper-limb dystonia (patients 1 and 3–6), characterised by dystonic posturing of the arms, and seven had recurrent episodic dystonic crises (patients 3–5, 7–9, and 11), characterised by severe generalised dystonia. In many, dystonic crises were managed at home by the parents (eg, with benzodiazepines), but sometimes needed hospital admission (patient 5). Patient 9 had episodic status dystonicus, with severe generalised dystonia, oculogyric crises, irritability, hyperthermia (either due to an intercurrent infection or secondary to status dystonicus), diaphoresis, marked rigidity, reduced consciousness, raised serum creatine kinase, severe rhabdomyolysis, and abnormal liver function tests. These episodes lasted up to several hours, and required hospital admission and sometimes intensive care treatment.

Parkinsonian features eventually became evident in all children. Six had generalised bradykinesia from disease onset (patients 1–6), and four developed this feature 1–30 months after presentation (patients 7–9 and 11). Generalised rigidity was a presenting feature in four children (patients 1, 3, 4, and 10), and developed in a further six patients within 1 year of symptom onset (patients 2, 5–7, 9, and 11). Cogwheeling rigidity was evident in four patients (patients 1–3 and 6). All children developed hypomimia, at a mean of 7 months (range 2–18) after presentation. A coarse, predominantly resting distal tremor was noted in eight children, three at presentation (patients 1, 3, and 4) and five at 2–36 months after presentation (patients 2, 5–7, and 9). Although the dystonia and rigidity complicated the assessment of resting tone, none of the children had pyramidal tract features at presentation. However, eight children (patients 1–7 and 9) developed pyramidal signs in early childhood of sustained ankle clonus, increased adductor tone, and increased four-limb hypertonicity when asleep. So far, three children younger than 5 years (patients 8, 10, and 11) have had no signs of pyramidal tract abnormality.

Axial hypotonia was noted at presentation in eight patients (patients 1–5 and 8–10), and developed within 1 year of clinical presentation in all other children (patients 6, 7, and 11). Eight children developed an eye movement disorder with ocular flutter, three at presentation (patients 4, 8, and 10) and five within 4–44 months after disease onset (patients 3, 5–7, and 9), which was confirmed by electronystagmography in patients 4 and 7. Six of these patients had saccade initiation failure and slow saccadic eye movements (patients 3–7 and 10), and two had eyelid myoclonus (patients 3 and 7). Oculogyric crises were common in patients 8 and 9.

The disease course affects motor and cognitive development, and is associated with secondary medical complications and reduced life expectancy. All children had severe gross motor delay. Progressive dystonia resulted in severe fixed postural dystonia, particularly affecting the upper limbs. Parkinsonian symptoms also progressed, resulting in akinesia, severe rigidity, obvious facial hypomimia, and prominent tremor. Although the presence of diurnal variation was difficult to assess in infants and young children, all patients older than 3 years did not show diurnal fluctuation of extrapyramidal symptom severity. Despite severe motor impairment, cognitive skills did not seem to be so severely affected. None of the children was able to speak, but despite absent expressive language, attributed mainly to motor deficit, most children had good receptive language and situational understanding. Many children developed methods of non-verbal communication, such as head and eye pointing techniques, and communication aids using eye gaze (eg, Tobii device). Formal neuropsychological assessment in patient 3 at age 11·3 years showed cognitive sparing relative to the patient's severe motor impairment (webappendix p 4). All children developed gastrointestinal complications, such as gastro-oesophageal reflux, constipation, excessive drooling, choking, feeding difficulties, and failure to thrive. Sleeping difficulties, orthopaedic complications, and frequent pneumonias were also prevalent. Four children (patients 4–7) have died (age range 8·9–16·2 years) secondary to respiratory complications and cardiac failure.

Although physicians attempted several drug treatments (including muscle relaxants, and dopaminergic, anticholinergic, antiglutaminergic, and γ-aminobutyric acid [GABA]-ergic agents), and surgical intervention (deep brain stimulator in patient 3), most treatments were either ineffective or provided a partial or temporary clinical response. Nine patients (patients 1–6 and 9–11) received levodopa combined with carbidopa (cocareldopa), with maximum doses of 2–6 mg/kg per day for a minimum of 2–3 weeks. Seven children (patients 1–6 and 10) had no clinical response, and treatment was limited by intolerable side-effects in four children (patients 4–6 and 10). In patients 9 and 11, doses of about 2 mg/kg per day improved upper-limb motor function and facial expression, and abolished episodic dystonic crises, but the dose could not be further increased because both patients developed drug-induced dyskinesias at higher doses. Subsequent addition of the dopamine agonist ropinirole in these patients led to further improvement of motor symptoms. After genetic diagnosis, ropinirole treatment was started in patient 3 at age 10·5 years, leading to reduced parkinsonian features, increased facial expression, an improved feeding pattern, and a mild reduction in upper-limb bradykinesia. The dopamine agonist pramipexole was ineffective in patient 4 at age 6 years and patient 10 at age 1·5 years, but resulted in some clinical improvement of bradykinesia and dystonia in patient 8 from age 15 months onwards.

CSF neurotransmitter analysis showed that all children had a raised ratio of homovanillic acid to 5-hydroxyindoleacetic acid of range 5·0–13·2 (normal range about 1·3–4·0; webappendix p 5). Excretion of urine homovanillic acid was slightly increased in six of seven children investigated, with range of 22–47 μmol homovanillic acid per mmol creatine (normal range 2–15 μmol homovanillic acid per mmol creatine). Serum prolactin was measured in seven patients (patients 1–4 and 7–9), and was raised in patients 2 and 3. Raised activity of serum creatine kinase, with further increase during dystonic crises, was recorded in six children, with range of 242–1773 IU/L (normal range 24–173 IU/L).

**Figure 1:**
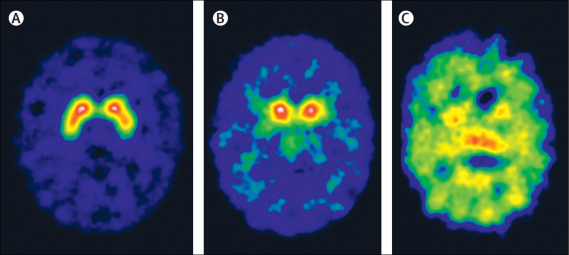
Nuclear brain imaging with single photon emission CT DaTSCAN in a control patient (A), a patient with juvenile parkinsonism of unknown aetiology (B), and patient 3 (C) Patients were age-matched. (A) Normal distribution of dopamine transporter sites. (B) Bilateral symmetrical loss of dopamine transporter sites from lentiform nuclei. (C) Complete loss of dopamine transporter sites in the basal nuclei, resulting in high background counts without any activity from basal nuclei.

After genetic diagnosis, DaTSCAN imaging in patient 3 at age 10 years showed complete loss of DAT activity in the basal nuclei, with high background counts on the image (figure 1). Although few paediatric data are available for comparison, the high background counts recorded have also been noted in adult patients with poor uptake in the basal nuclei, and could be attributed to unbound DaTSCAN in the blood pool (TL, personal communication). Loss of DAT activity in patient 3 was more extensive than in a patient aged 9·2 years with juvenile parkinsonism of unknown aetiology (not dopamine transporter deficiency syndrome) who had symmetrical reduction of DAT activity in the basal nuclei (figure 1). On brain MRI, no patients had gross structural defects or signal abnormalities in the basal ganglia. Eight children (patients 1, 3, 4, and 6–10) had subtle neuroradiological abnormalities, such as prominence of the external frontotemporal subarachnoid spaces and mild delay in myelination. Patient 9 had a white matter abnormality similar to periventricular leucomalacia (webappendix p 6). Brain MRS in three children (patients 1, 2, and 11) did not show any abnormalities.

*SLC6A3* gene mutations were identified in all patients (table 2). Mutations identified in patients 1–3 have been reported previously,^9^ and novel *SLC6A3* mutations were detected in patients 4–11. No mutation hotspots were noted. Within each patient's family, identified mutations segregated appropriately with dopamine transporter deficiency syndrome disease status (parents obligate heterozygote carriers, unaffected siblings either wild type or carriers). None of the identified mutations was reported as a polymorphism in genomic databases or was detected from analysis of more than 300 control chromosomes matched for ethnic origin. Sequence alignment for novel missense mutations showed Val158, Gly327, Arg521, Pro529, and Pro554 to be highly conserved throughout vertebrate species, and Leu224 to be highly conserved throughout mammalian species (webappendix p 7). The deletion and nonsense mutations were predicted to result in nonsense-mediated decay (and no expression of DAT), or a truncated protein product. Patients 4 and 6 were homozygous for splice variants predicted to cause aberrant splicing according to the Berkeley Drosophila Genome Project's Splice Site Prediction. In patient 8, exons 12 and 13 failed to amplify on repeated occasions, suggesting a homozygous deletion. Long-range PCR was used to characterise the putative deletion and define the genomic deletion breakpoint (webappendix pp 8–9).

**Table 2:** SLC6A3 mutations identified in patients with dopamine transporter deficiency syndrome

	**Ethnic origin**	**Parental consanguinity**	**Mutation status**	**Mutations in DNA**	**Exon**	**Effect on protein product**
Patient 1	Pakistani	Yes	Homozygous	1103T→A	8	Leu368Gln
Patient 2	Pakistani	Yes	Homozygous	1103T→A	8	Leu368Gln
Patient 3	Mixed European descent	Yes	Homozygous	1184C→T	9	Pro395Leu
Patient 4	Mixed European descent	No	Homozygous	1156+5delG	Intron 8 splice site	Unknown
Patient 5*	Mixed European descent	No	Compound heterozygous	472G→T	4	Val158Phe
				1661C→T	13	Pro554Leu
Patient 6	Turkish	Yes	Homozygous	1031+1G→A	Intron 7 splice site	Unknown
Patient 7*	Mixed European descent	Yes	Homozygous	399delG	3	Ile134SerfsX5
Patient 8	Mixed European descent	No	Homozygous	1499_1767del	12, 13	Gly500GlufsX13
Patient 9	Mixed European descent	No	Compound heterozygous (three variants)	979G→A	7	Gly327Arg
				1315C→T	10	Gln439X
				1586C→T	12	Pro529Leu
Patient 10	Mixed European descent	Yes	Homozygous	671T→C	5	Leu224Pro
Patient 11	Mixed European descent	Yes	Homozygous	1561C→T	12	Arg521Trp

* DNA not available for proband who had died some years before the study; both parents were heterozygous for the described mutations.

For all identified missense mutations, the transport activity of mutant hDAT proteins was compared with that of wild-type hDAT (webappendix p 10). Wild-type hDAT had normal transport activity, but the mutant proteins showed either non-specific uptake (Leu368Gln, Pro395Leu, Val158Phe, Pro554Leu, Gly327Arg, Gly327Arg plus Gln439X, and Leu224Pro) or lower uptake than that of wild-type hDAT (6% of wild-type activity for Pro529Leu, and 27% of wild-type activity for Arg521Trp). Coexpression of Gly327Arg plus Gln439X with Pro529Leu (as in patient 9) further lowered activity to non-specific uptake. The K_d_ of ^3^H-CFT was near normal in the mutants at 13–44 nM (17 nM in wild type) apart from in the Gly327Arg plus Gln439X mutant, for which binding was substantially lower, with a higher K_d_ of 101 nM. In many mutants, non-specific binding of ^3^H-CFT was higher than in wild type, contributing to greater than normal variation in the data. The K_i_ of dopamine in inhibition of cocaine-analogue binding was near that of wild-type hDAT in the mutants Pro395Leu, Pro554Leu, Gly327Arg, Pro529Leu, and Arg521Trp, but was reduced (higher K_i_) in the Leu368Gln mutant and, to a lesser extent, in the Gly327Arg plus Gln439X and Leu224Pro mutants. Maximal binding of ^3^H-CFT to cells, mainly representing surface binding,^15^ indicated decreases of three–five times in mutants Leu368Gln, Pro554Leu, Gly327Arg plus Gln439X, Pro529Leu, and Arg521Trp, and, to a lesser extent, in the Pro395Leu mutant, whereas decreases of 15 times or more were recorded in the mutants Val158Phe, Gly327Arg, and Leu224Pro (webappendix p 10).

**Figure 2:**
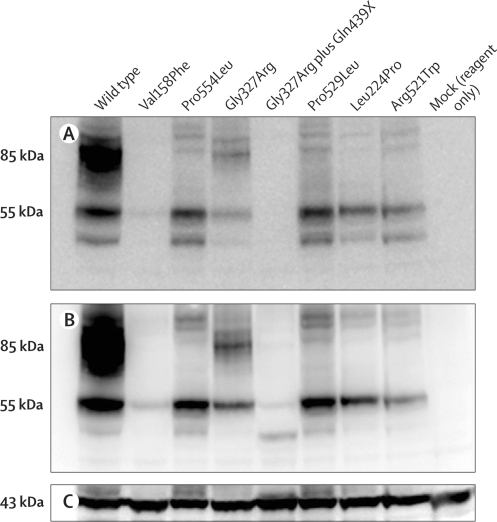
Expression of wild-type and mutant human dopamine transporter in HEK293 cells Protein was probed with antibodies against the C-terminal (A) and N-terminal (B) of the dopamine transporter, and with anti-β-actin antibody to show the relative equivalent loading of total protein (C). The same amount of total lysate protein was loaded in all lanes. The mutations not reported in previous studies are shown in the same sequence as in webappendix p 10, starting with patient 5.

Analysis of whole cell lysates by immunoblotting with anti-C-terminal DAT antibody showed deficiency in mature DAT (80 kDa) in the novel mutants Val158Phe, Pro554Leu, Pro529Leu, Leu224Pro, and Arg521Trp (figure 2A), and in the previously studied mutants Leu368Gln and Pro395Leu.^9^ By contrast, Gly327Arg resembled wild type with a stronger band of mature DAT than in the other mutants. As expected, the Gly327Arg plus Gln439X mutant with the C-terminal deleted did not show a signal with anti-C-terminal DAT antibody (figure 2A), but some expression of this mutant was detected with anti-N-terminal DAT antibody in the form of low-molecular-weight bands (figure 2B). For all other mutants, the patterns recorded with anti-terminal DAT antibody (figure 2B) were identical to those recorded with antibody to the C-terminal. The Val158Phe mutant had particularly low expression.

## Discussion

We report a detailed description of a precisely diagnosed series of children with autosomal recessive dopamine transporter deficiency syndrome, a complex movement disorder, which we have shown is associated with loss-of-function mutations in the *SLC6A3* gene (panel). Dopamine transporter deficiency syndrome is thus a dopamine transportopathy and is the first identified disorder with parkinsonian features due to genetic alterations of DAT.^6^ Because inherited diseases are often named after the underlying pathogenic defect, we now refer to this disorder as hereditary dopamine transporter deficiency syndrome as an indication of the underlying disease mechanism and to encompass the range of clinical phenotypes.

Panel **Research in context**

**Systematic review**

In 2009, we identified *SLC6A3* mutations in three children with infantile parkinsonism-dystonia.^9^ We searched PubMed and could not find a similar study characterising this autosomal recessive genetic disorder.

**Interpretation**

Detailed clinical and molecular analysis of a cohort of children with dopamine transporter deficiency syndrome has allowed comprehensive delineation of this newly recognised clinical disorder. Our findings confirm that hereditary dopamine transporter deficiency syndrome is a novel neurotransmitter disorder caused by loss-of-function mutations in the gene encoding the dopamine transporter (*SLC6A3*), resulting in an early-onset severe motor disorder.

In this study, we showed loss of DAT function in dopamine transporter deficiency syndrome. All mutations that were predicted to result in expression of hDAT protein severely incapacitated dopamine transport. Mechanisms underlying the loss of DAT transport function include reduced expression of DAT, loss of dopamine recognition by DAT due to reduced binding affinity, and lack of glycosylation to form mature DAT, which is known to negatively affect trafficking to the cell surface and transport function of DAT.^16^ Homology modelling of these point mutants, based on the structure of the bacterial analogue LeuT,^17^ shows subtle structural changes compared with wild type. These changes are far distal from the mutated residue and could affect conformational changes needed during dopamine transport (data not shown). The findings of in-vivo DaTSCAN imaging in patient 3 are consistent with the in-vitro assessment of DAT function, providing further evidence that loss of DAT function has a causative role in the pathogenesis of dopamine transporter deficiency syndrome.

In dopamine transporter deficiency syndrome, we postulate that defective reuptake of dopamine into the presynaptic neuron causes accumulation of extraneuronal dopamine, thus resulting in dopamine degradation^6^ and raised concentrations of homovanillic acid in CSF neurotransmitter analysis. Dopamine transporter defects do not affect the serotonin biosynthetic pathway and concentrations of 5-hydroxyindoleacetic acid in CSF are normal in dopamine transporter deficiency syndrome, leading to an increased ratio of homovanillic acid to 5-hydroxyindoleacetic acid in CSF. Poor dopamine reuptake leads to depleted presynaptic stores of intracellular dopamine to be packaged into synaptic vesicles for release extraneuronally with perisynaptic diffusion.^6^, ^18^ Excess extraneuronal dopamine might also overstimulate presynaptic D_2_ autoreceptors (D_3_ receptors), resulting in inhibition of tyrosine hydroxylase and thereby decreasing dopamine production.^6^ Excess extraneuronal dopamine might additionally have postsynaptic effects, such as downregulation or desensitisation of postsynaptic dopamine receptors, with alterations in downstream signalling. Many of these physiological consequences of transporter dysfunction are evident in mutant mice lacking DAT and other monoamine transporters.^19^

**Figure 3:**
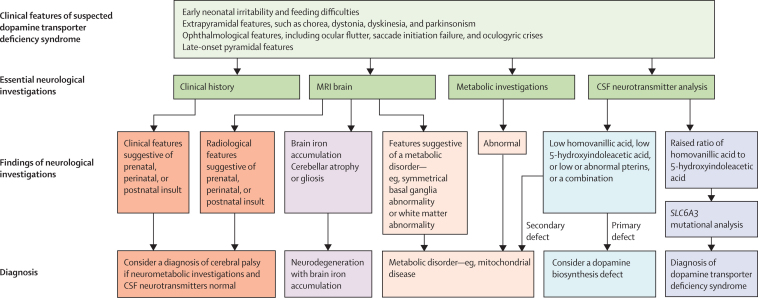
Diagnostic algorithm for dopamine transporter deficiency syndrome and differential disorders CSF=cerebrospinal fluid. Neurometabolic investigations need to be tailored according to the clinical presentation, but could include serum assessment of lactate, ammonia, biotinidase, carnitine, acylcarnitine profile, aminoacids, thyroid function tests (including free triiodothyronine); urine assessment of organic acids, aminoacids, purines, and pyrimidines; and CSF assessment of lactate, glucose, aminoacids, and protein. Assessment of a muscle or skin biopsy sample, specialist metabolic tests, and genetic investigation might also be appropriate. A CSF neurotransmitter profile should include homovanillic acid, 5-hydroxyindoleacetic acid, and pterins.^5^, ^21^

Many patients with dopamine transporter deficiency syndrome are likely to be undiagnosed. In many centres, CSF neurotransmitter analysis might not be a routine investigation for children with a complex motor disorder.^20^ Furthermore, because dopamine transporter deficiency syndrome is rare and some of its clinical features are present in several infantile neurological syndromes, a definitive diagnosis cannot be made on clinical grounds alone. The clinical presentation of dopamine transporter deficiency syndrome can mimic dyskinetic, spastic, and mixed cerebral palsy. The differential diagnosis of dopamine transporter deficiency syndrome also includes disorders of dopamine biosynthesis,^21^ neurodegenerative disorders, such as neurodegeneration with brain iron accumulation,^22^ and early-onset neurometabolic syndromes, such as mitochondrial diseases.^23^ For accurate diagnosis of dopamine transporter deficiency syndrome, the key clinical features need to be recognised and followed by appropriate biochemical and genetic investigations (figure 3).

The features of dopamine transporter deficiency syndrome seem to result from dopamine dysregulation, which might eventually result in cerebral dopamine deficiency.^21^, ^24^, ^25^ Several other infantile neurotransmitter disorders caused by enzyme defects in the pterin and dopamine biosynthetic pathway also result in dopamine deficiency (table 3).^5^, ^21^ Therefore, the fact that dopamine transporter deficiency syndrome shares many clinical features reported in such disorders is not surprising, but dopamine transporter deficiency syndrome and dopamine biosynthesis disorders also have distinct clinical, biochemical, and therapeutic differences (table 3). Most importantly, the raised ratio of homovanillic acid to 5-hydroxyindoleacetic acid in CSF does not occur in any other disorder of dopamine metabolism and is therefore a key finding for the diagnosis of dopamine transporter deficiency syndrome.

**Table 3:** Comparison of dopamine transporter deficiency syndrome with infantile neurotransmitter disorders of dopamine biosynthesis

		**Dopamine transportopathy**	**Dopamine biosynthesis defect**
Clinical syndrome		Dopamine transporter deficiency syndrome	Autosomal recessive GTP cyclohydrolase deficiency; autosomal dominant GTP cyclohydrolase deficiency (classically milder); pyruvoyl-tetrahydropterin synthase deficiency; sepiapterin reductase deficiency; dihydropteridine reductase deficiency; tyrosine hydroxylase deficiency; or aromatic acid decarboxylase deficiency
Age of onset		Infancy	Usually infancy (rarely after the infantile period)
Clinical features			
	Early irritability	Yes	Yes
	Feeding difficulties, swallowing difficulties	Yes	Yes
	Bulbar dysfunction	Yes	Yes
	Global developmental delay	Yes	Yes
	Truncal hypotonia	Yes	Yes
	Prominent extrapyramidal symptoms (dystonia, choreoathetosis, parkinsonism, tremor, and oculogyric crises)	Yes	Yes
	Autonomic features (hyperthermia, sweating, hypersalivation, sleep disturbance)	Yes	Yes
	Pyramidal tract features	Yes	Yes
	Diurnal variation of symptoms	Not reported in children older than 3 years (in whom diurnal variation would be assessable)	Might be evident (eg, GTP cyclohydrolase deficiency)
	Microcephaly	Not often reported	Reported
	Seizures	Not reported	Reported
Ocular features			
	Oculogyric crises	Reported in a few patients	Often reported
	Ocular flutter and saccade initiation failure	In most patients	Not reported
Dystonic storms or status dystonicus		Often reported	Rarely reported
CSF neurotransmitter analysis		Raised ratio of homovanillic acid to 5-hydroxyindoleacetic acid	Reduced homovanillic acid, reduced 5-hydroxyindoleacetic acid, or abnormal pterins, or a combination
Clinical response to therapeutic agents		Partial and temporary	Can be substantial in some dopamine biosynthesis defects (eg, levodopa in tyrosine hydroxylase deficiency and GTP cyclohydrolase deficiency)

CSF=cerebrospinal fluid.

Therapeutic strategies have either little or no effect on the clinical symptoms of dopamine transporter deficiency syndrome, unlike some dopamine biosynthesis disorders. Notably, the two patients who showed a clinical response to cocareldopa (patients 9 and 11) had mutations that were shown to be associated with some residual DAT activity on functional investigation. Characterisation of further cases of dopamine transporter deficiency syndrome will allow improved interpretation of whether genotype can predict phenotype (disease severity or response to drug treatment). In this study, use of the dopamine receptor agonists ropinirole and pramipexole (used in adult-onset Parkinson's disease^26^) in patients with dopamine transporter deficiency syndrome had some benefit, but the optimum paediatric dose, side-effect profile in the paediatric population, and effect on long-term outcome need to be established. Future neuromodulatory therapeutic strategies, with dopamine agonists and deep brain stimulation,^27^ might need to be used early in the disease course before the postulated compensatory changes in postsynaptic signalling pathways have occurred.

We have delineated the clinical, molecular genetic, and functional features of a novel disorder. Increased recognition of dopamine transporter deficiency syndrome will expand the range of phenotypes identified, allow accurate diagnosis and genetic counselling, prevent unnecessary investigations, provide further insights into genotype–phenotype correlations and DAT function, and accelerate the development of therapeutic strategies.

## Web Extra Material

Supplementary webappendix

Webvideo 1 Generalised hyperkinesia and orolingual dyskinesia (patient 10)

Webvideo 2 Generalised dyskinesia (patient 7)

Webvideo 3 Dystonia (patients 8 and 4)

Webvideo 4 Bradykinesia and dystonia (patient 8)

Webvideo 5 Parkinsonian and dystonic features (patient 4)

Webvideo 6 Tremor and dystonia (patient 4)

Webvideo 7 Parkinsonian and dystonic features (patient 7)

Webvideo 8 Eye movement disorder (patients 8, 4, and 7)
