# Supplementary material for: Clinical and molecular characterisation of hereditary dopamine transporter deficiency syndrome: an observational cohort and experimental study
Source: Lancet Neurol. 2010 Nov 26;10(1):54–62. doi: 10.1016/S1474-4422(10)70269-6 (PMC3002401; doi:10.1016/S1474-4422(10)70269-6)
Supplement: Supplementary webappendix [file mmc1.pdf]

THE LANCET Neurology

Supplementary webappendix

This webappendix formed part of the original submission and has been peer reviewed. We post it as supplied by the authors.

Supplement to: Kurian MA, Li Y, Zhen J, et al. Clinical and molecular characterisation of hereditary dopamine transporter deficiency syndrome: an observational cohort and experimental study. *Lancet Neurol* 2010; published online Nov 26. DOI:10.1016/S1474-4422(10)70269-6.

WEB APPENDIX

Metabolic pathway of serotonin and dopamine biosynthesis

Metabolic pathway of serotonin and dopamine biosynthesis (solid arrows) and catabolism (broken arrows). Tetrahydrobiopterin (BH_4) is the essential cofactor for the rate-limiting enzymes tryptophan hydroxylase and tyrosine hydroxylase (TH). BH_4 is synthesised de novo via steps 8-11and recycled via the salvage pathway (steps 12-13).

GTP is guanosine triphosphate, **H_2NP_3** is dihydroneopterin triphosphate, **NEO** is neopterin, **6-PTP** is 6-pyruvoyltetrahydropterin, **SPT** is sepiapterin, **BH_4** is tetrahydrobiopterin and q**BH_2** is (quinonoid) dihydrobiopterin, **5-HTP** is 5 hydroxytryptophan, **L-DOPA** is laevodihydroxyphenylalanine (laevodopa), **OMD** is 3-ortho-methyldopa, **VLA** is vanillyllactic acid, **5-HIAA** is 5-hydroxyindoleacetic acid, **HVA** is homovanillic acid.

1 is tryptophan hydroxylase, **2** is tyrosine hydroxylase (**TH**), **3** is aromatic L-amino acid decarboxylase (**AADC**), **4** is monoamine oxidase (**MAO**)**, 4a** is monoamine oxidase plus aldehyde dehydrogenase**, 5** is catechol-*O*-methyltransferease (**COMT**)**, 6** is dopamine β-hydroxylase,**7** phenylethanolamine N-methyltransferase, **8** is GTP-cyclohydrolase I (**GTPCH I**), **9** is 6-pyruvoyltetrahydropterin synthase (**PTPS**)**, 10** is aldose reductase, **11** is sepiapterin reductase (**SR**), **12** is pterin-4α-carbinolamine dehydratase**, 13** is dihydropteridine reductase (**DHPR**)**.**

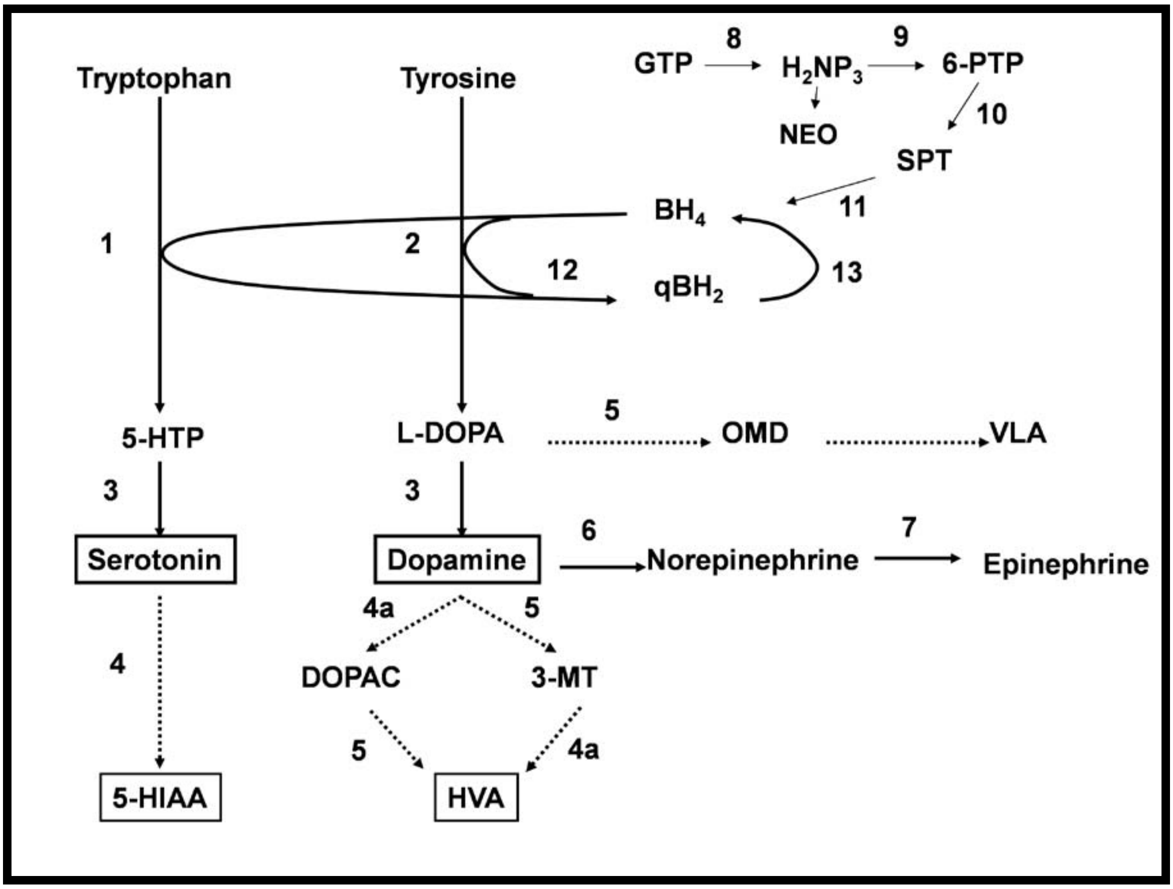

Long Range PCR Techniques For Determination of the Genomic Deletion Breakpoint

A long range PCR kit (PCR Extender System, Flowgen Bioscience, Nottingham, UK) was used for PCR amplification. Long range PCR conditions consisted of an initial denaturation step at 93ºC for 5 min followed by 35 cycles of 45s denaturation at 93ºC, 45s annealing at 60ºC and 8 min extension at 68ºC with a final extension at 68ºC for 5 min. The PCR products were either gel-extracted or purified with MicroCLEAN (Web Scientific, Crewe, UK), directly sequenced by Big Dye Terminator Cycle Sequencing System (Applied Biosystems, Foster City, USA) and then cleaned up using the EDTA (ethylenediaminetetracetic acid) method of precipitation. Sequencing reactions were run on an ABI PRISM 3730 DNA Analyzer (Applied Biosystems) and then analyzed using Chromas software (http://www.technelysium.com.au/chromas.html).

Primers used for site-directed mutagenesis (IDT, Inc., Coralville, IA, USA)

**Mutant**	**Primers (5’ to 3’)**
Val158Phe	TC TTT TAC AAC TTC ATC ATT GCC TG
Leu224Pro	CGG GGC GTG CTG CAC CCC CAT CAG AGC CAC GGC
Gly327Arg	GGC GTG GGA TTC AGG GTC CTG ATC GCC TTC
Arg521Trp	CCA GCC TGT ACT GGT GGCTGT GCT GGA AGC
Pro529Leu	TGG AAG CTC GTC TCC CTC TGC TTC CTC CTG TTC
Pro554Leu	GGC GCC TAC ATC TTC CTC GAC TGG GCC AAT GCA
Gln439X - Gly327Arg	CTG ATC GAC GAA TTC TAG CTC CTG CAC CGG CAT

Neuropsychology Assessment of Patient 3

Patient 3 had a formal neuropsychology examination at age 11.3 years. Despite the extensive physical disability, formal assessment demonstrated that:

- She could effectively use a communication book comprising four pictures for judgements via eye gaze.
- She also demonstrated good working memory in response to verbal questions and prompted when given alternatives or choices.
- She was able to effectively mobilise (in response to verbal commands) via the use of head switches on her electric wheelchair.
- She exhibited good social awareness and empathy as demonstrated by her enjoyment of watching the news or age-appropriate films and her ability to identify distressing situations when these are embedded within a complex narrative.
- She demonstrated accurate recognition of familiar information.

CSF neurotransmitter findings in the DTDS cohort

Patient	Age at Lumbar Puncture (Years)	CSF HVA Concentration (nmol/L)	CSF HVA Reference Range[A] (nmol/L)	CSF HIAA Concentration (nmol/L)	CSF HIAA Reference Range[A] (nmol/L)	CSF HVA:HIAA Ratio[B]
1	3.5	1873	154 - 867	141	89 - 367	**13.2**
2	1.3	1704	154 - 867	250	89 - 367	**6.8**
3	2.5	1135	154 - 867	91	89 - 367	**12.5**
4	3.7	2046	384 -769	169	110 – 265	**12.1**
	4.0	1914	384 -769	165	110 - 265	**11.6**
	8.9	1099	285 - 560	131	101 - 237	**8.4**
5	5.0	1633	154 - 867	143	89 - 367	**11.4**
6	3.0	1810	154 - 867	149	89 - 367	**12.1**
7	7.5	1043	346 – 716	158	100 - 245	**6.6**
	8.3	814	339 – 668	143	109 - 214	**5.7**
8	0.5	2048	310 - 1100	238	150 - 800	**8.6**
	0.75	1114	403 – 919	225	170 - 412	**5.0**
9	1.5	2280	294-1115	176	129-520	**12.9**
	2.5	1345	233-928	176	74-345	**7.7**
10	0.8	3000	295 - 932	285	114 – 336	**10.5**
11	4.2	1600	233-928	145	74-345	**11.0**

A Age-related HVA and HIAA reference ranges were established by each laboratory according to laboratory-specific sampling protocols.[10,11,12]

B The HVA concentration/HIAA concentration is stable throughout the craniocaudal gradient and does not change significantly with age. Published data suggests that the normal range (above 4 months of age) is approximately 1.3–4.0[10,11,12]

MRI Brain

Patient 9, Age 1.2 years.

Axial T1-weighted images indicating mild hyperintensity of the periventricular white matter (yellow arrows)

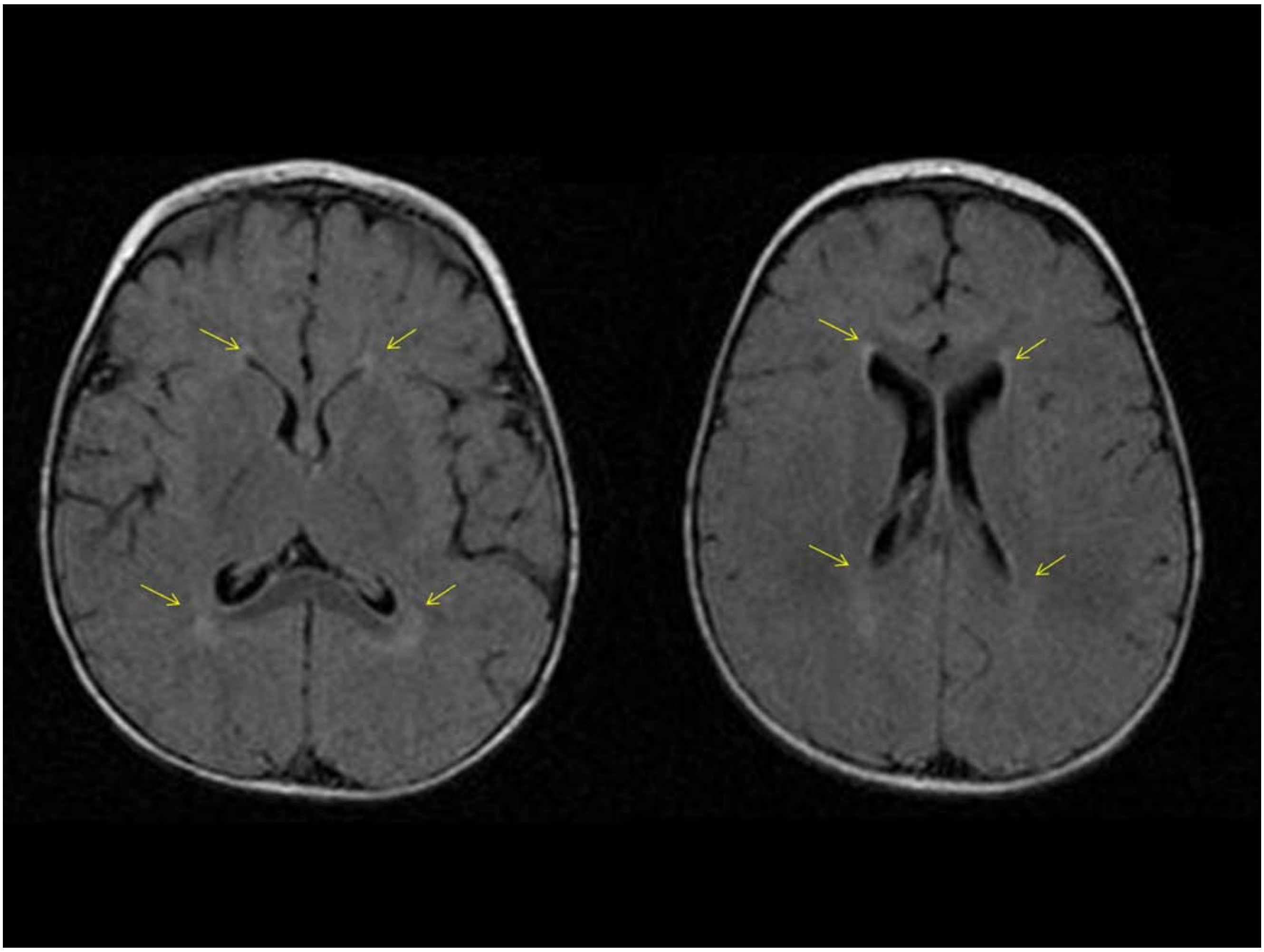

<u>Conservation of the mutated *SLC6A3* residues</u>

Conservation of the mutated *SLC6A3* residues V158, L224, G327, R521, P529, P554. Six representative vertebrate sequences are aligned.

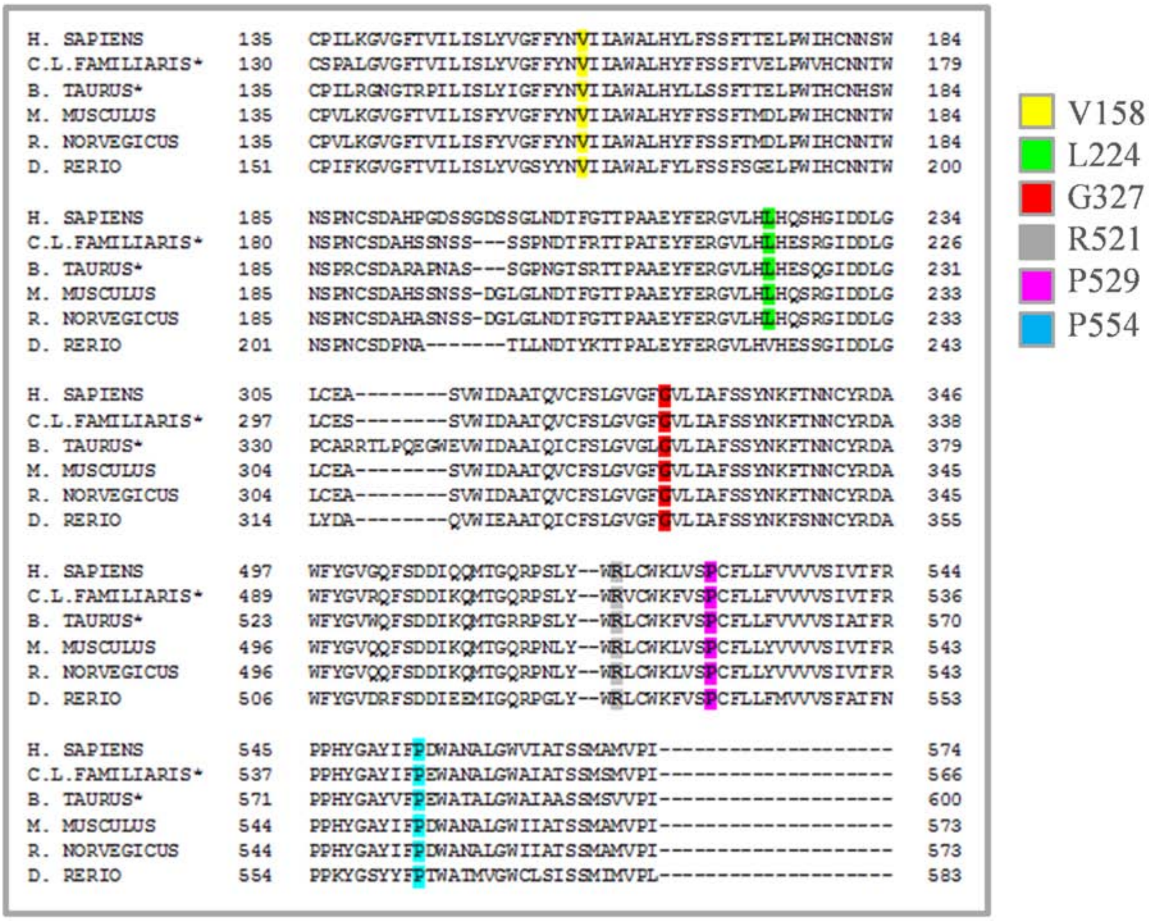

* Predicted protein sequence

Characterisation of the genomic breakpoint of the homozygous deletion in patient 8

A forward primer in intron 10 (FP) and a reverse primer in intron 14 (RP) were utilised for long range PCR

Primers utilised for delineation of the breakpoint in Patient 8

Primer	Location (Bp)	Primer Sequence
FP	1,462,303–1,462,320	GTGTGCACAGTGAATCCC
RP	1,453,989–1,453,970	CTGAGCTTGGGATCATTCTG

An amplicon was obtained both in the index case and mother but no PCR product was amplifiable in 400 ethnically matched control chromosomes (webappendix p9). Sequencing of this PCR fragment (using serial reverse primers in intron 13) was undertaken.

Primers utilised for delineation of the breakpoint in Patient 8

Primer	Location (Bp)	Primer Sequence
DAT_13.1R	1,454,057–1,454,176	CCACCACTGACTCACACTGC
DAT_13.2R	1,454,401–1,454,419	CCTGGAGATGGCTCTTGAG
DAT_13.3R	1,454,710–1,454,728	CACTTGTACCTGGCTCAGC
DAT_13.4R	1,454,940–1,454,961	CATCAGCTTTCCTGGGGCCATG
DAT_13.5R	1,455,276–1,455,295	GGAGTGTCCTTGTCTCTTCC
DAT_13.6R	1,455,556–1,455,575	CAAACACTTGGACACGAATG
DAT_13.7R	1,455,790–1,455,808	CACACACACAGTGTTGTGG

The genomic deletion was thus characterised (webappendix p9). The telomeric and centromeric genomic breakpoints were mapped to 1,455,093–1,455,095bp (intron 13) and 1,460,244-1,460,246bp (intron 11) respectively, defining a ~5kb deletion The precise location of the breakpoint could not be further defined as there was a 3 bp sequence (CAG) common to both the intron 11 and intron 13 sequence (webappendix p9). The mechanism by which the deletion was generated is unclear. Neither the centromeric nor telomeric deletion breakpoints occurred within a repetitive sequence, or within regions of sequence homology (http://www.repeatmasker.org).

<u>Definition of the *SLC6A3* deletion in Patient 8.</u>
<u>A: Agarose gel photograph of PCR amplification</u>
PCR amplification of introns 10 to 14 of *SLC6A3* showing the presence of bands in Patient 8 (Lane 5) and their parent (Lane 6) and the absence of bands in normal controls (Lanes 1-4). Lane 7 is a negative control.
<u>B: Determination of the DNA sequence of the genomic breakpoint of the deletion</u>
The exons that are present are indicated in red (exon 11 and 14). The deleted introns and exons are represented in grey (exon 12, intron 12 and exon 13). The telomeric and centromeric genomic breakpoints were mapped to 1,455,093–1,455,095bp (intron 13, shaded in blue) and 1,460,244-1,460,246bp (intron 11, shaded in blue) There is a 3 base region of homologous sequence which show 100% homology in both intron 11 and 13 (shaded yellow box).

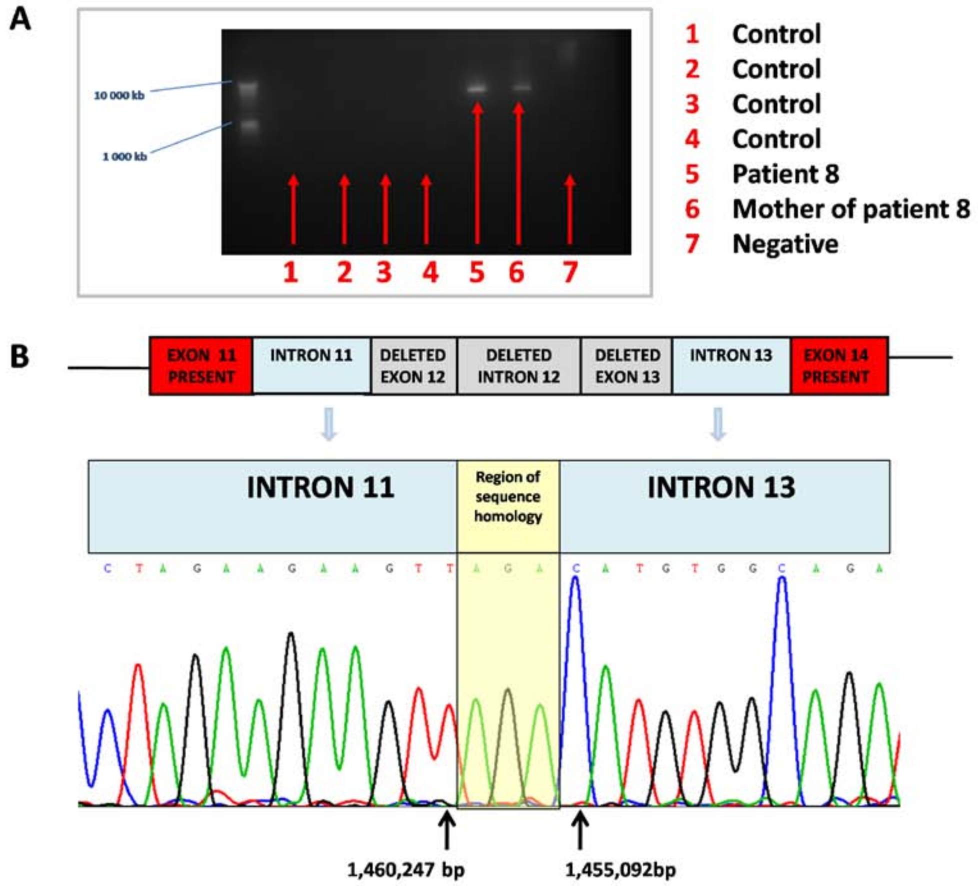

Dopamine transport and cocaine analog binding by wild-type and mutant hDAT

Patient	hDAT Mutant	[^{3}H]dopamine uptake	[^{3}H]CFT binding		
			K_d (nM)	B_{max} (pmole/mg)	Inhibition by dopamine, K_i (µM)
	Wild-type	Normal	17.2 $\pm$ 1.7	2.83 $\pm$ 0.73	5.38 $\pm$ 0.67
1	L368Q [A]	0[C]	35.6 $\pm$ 8.2	0.384 $\pm$ 0.110 *	52.9 $\pm$ 1.7 *
2	L368Q [A]	0[C]	35.6 $\pm$ 8.2	0.384 $\pm$ 0.110 *	52.9 $\pm$ 1.7 *
3	P395L [A]	0[C]	30.2 $\pm$ 6.5	0.962 $\pm$ 0.144	3.35 $\pm$ 0.65
4	- [B]				
5	V158F<br>P554L	0[C]<br>0[C]	N/A[D]<br>43.8 $\pm$ 5.9	N/A[D]<br>0.48 $\pm$ 0.05 *	N/A[D]<br>4.39 $\pm$ 0.71
6	- [B]				
7	- [B]				
8	- [B]				
9	G327R<br>G327R-439X<br>P529L<br>G327R-439X with P529L	0[C]<br>0[C]<br>6.2 $\pm$ 1.3% of WT[E]<br>1.7 $\pm$ 1.4% of WT[E]	24.3 $\pm$ 12.4<br>101 $\pm$ 6 *<br>16.5 $\pm$ 5.4	0.18 $\pm$ 0.04 *<br>0.85 $\pm$ 0.14 *<br>0.47 $\pm$ 0.07 *	2.93 $\pm$ 1.59<br>28.1 $\pm$ 21.6<br>3.54 $\pm$ 1.75
10	L224P	0[C]	13.0 $\pm$ 1.3	0.15 $\pm$ 0.04 *	28.8 $\pm$ 6.0
11	R521W	26.9 $\pm$ 2.9% of WT[E]	20.5 $\pm$ 3.1	0.48 $\pm$ 0.09 *	3.82 $\pm$ 1.37

[A] Data from reference 9
[B] Protein product unknown or frameshift
[C] No transport activity above nonspecific uptake
[D] No binding detectable above nonspecific binding
[E] P<0.002 compared with wild-type (one-sample t-test with wild-type at 100%). Average uptake in WT was 23 fmol/min/mg protein at 6 nM [^{3}H]dopamine in the assay
*** P<0.05 compared with wild-type (1-way ANOVA followed by Dunnett multiple comparisons with wild-type). Statistics were applied to mutant groups compared with wild-type examined within one and the same experimental set; wild-type values listed are the average of all experiments. There were two sets: controls (wild-type) along with mutants assayed for cases 1 through 3 (data from Kurian et al., 2009), and controls (wild-type) along with mutants assayed for cases 4 through 11 (with 3-5 independent determinations per group). For the former set, the wild-type values for K_d, B_{max}, and dopamine K_i were 15.9 $\pm$ 1.9, 2.14 $\pm$ 0.65, and 6.58 $\pm$ 0.53; the respective values for the latter set were 18.8 $\pm$ 3.2, 3.69 $\pm$ 1.44, and 4.87 $\pm$ 1.32. Data shown in Table are mean $\pm$ SEM for 8-9 (wild-type) or 3-5 (mutant) independent determinations per group**

VIDEO LEGENDS
VIDEO 1
Clip 1: Patient 10, age 17 months
Generalised hyperkinesia of all four limbs. Striatal toe (hyperextension of the big toe) is evident in the right foot.
Clip 2: Patient 10, age 8 months
Orolingual dyskinesia.
VIDEO 2
Patient 7, age 8 months
Clip 1: Patient in pram and Clip 2: Patient in high chair
Florid dyskinesia characterised by repetitive generalised choreiform movements of variable amplitude
VIDEO 3
Clip 1: Patient 8, age 9 months
Dystonic posturing of the left arm and right foot. Bilateral striatal toe is also evident.
Clip 2: Patient 8, age 15 months
Dystonic posturing of the left arm and right foot. Dystonia of the right arm and fingers is also evident when attempting to grasp the toy ring.
Clip 3: Patient 4, age 17 months
Prominent dystonia of the upper limbs and jaw. Lower limbs are also dystonic with some rigidity
VIDEO 4
Patient 8, age 2.1 years
Evidence of bradykinesia and dystonia of the left arm and fingers on attempting to grasp a toy.
VIDEO 5
Patient 4, age 4.5 years
Evolution of a parkinsonian phenotype. Patient 4 has developed paucity of spontaneous movement with bradykinesia and reduced facial expression. There is also evidence of dystonia (oromandibular dystonia and dystonic upper limb posturing).
VIDEO 6
Patient 4, age 8 years
A prominent coarse tremor is evident, especially in the upper limbs. Dystonic posturing of the jaw and upper limbs is also illustrated.
VIDEO 7
Patient 7, age 8.5 years
Progression of parkinsonian features with striking hypomimia, generalised akinesia and a coarse tremor of the left arm. Dystonic posturing of the right arm is also illustrated
VIDEO 8
Clip 1: Patient 8, age 16 months
Oculogyric crisis with both eyes fixating in downward gaze
Clip 2: Patient 4, age 4 years
Ocular flutter
Clip 3: Patient 7, age 8 years
Ocular flutter and bilateral eyelid myoclonus
Clip 4: Patient 4, age 8 years
Saccade initiation failure
